# A Sanitary Wash-House

**Published:** 1890-04

**Authors:** 


### A SANITARY WASH-HOUSE.

Albert Shaw has a most suggestive paper in the March *Century* entitled "Glasgow; a Municipal Study," from which we quote: "Not the least important feature of the health department's work in Glasgow is the Sanitary Wash-house. A similar establishment should be a part of the municipal economy of every large town. In 1864, the authorities found it necessary to superintend the disinfection of dwellings, and a small temporary wash-house was opened, with a few tubs for the cleansing of apparel, etc., removed from the infected houses. For a time after the acquisition of Belvidere, a part of the laundry of the hospital was used for the purpose of a general sanitary wash-house. But larger quarters being needed, a separate establishment was built and opened in 1883, its cost being about $50,000. This place is so admirable in its system and its mechanical appointments that I am again tempted to digress with a technical description. The place is in constant communication with sanitary headquarters, and its collecting wagons are on the road early every morning. The larger part of the articles removed for disinfection and cleansing must be returned on the same day, to meet the necessities of poor families. I visited the house on a day when 1,800 pieces, from 25 different families, had come in. In 1887, 6,700 washings, aggregating 380,000 pieces, were done. The quantity, of course, varys from year to year with the amount of infectious disease in the city. The establishment has a crematory, to which all household articles whatsoever that are to be burned after a case of infectious disease, must be brought by the vans to the sanitary department. The carpet cleaning machinery and the arrangements for disinfection by steam, by chemicals, and by boiling I cannot here describe.

"The department's disinfecting and whitewashing staff is operated from the wash-house as headquarters. A patient being removed to the hospital, the authorities at once take possession of the house for cleansing and disinfection. It is a point of interest also that the city has provided a comfortable 'house of reception' of some ten rooms, with two or three permanent servants, where families may be entertained for a day or more as the city's guests, if it is desirable to remove them from their homes during the progress of the disinfecting and clothes washing operations. The house is kept in constant use, and it is found a very convenient thing for the department to have at its disposal.

"As net results of the sanitary work of the Glasgow authorities may be mentioned the almost entire extinction of some of the worst forms of contagious disease, and a mastery of the situation, which leaves comparatively little fear of widespread epidemics in the future, in spite of the fact that Glasgow is a great seaport, has an unfavorable climate, and has an extraordinarily dense and badly housed working population. The steady decline of the total death-rate, and its remarkably rapid decline as regards those diseases at which sanitary science more especially aims its weapons, are achievements which are a proper source of gratification to the town council and the officers of the health department."

